# Right-sided endocarditis caused by polyclonal *Staphylococcus aureus* infection

**DOI:** 10.1186/s40001-021-00549-z

**Published:** 2021-08-11

**Authors:** Yotam Kolben, Yuval Ishay, Henny Azmanov, Assaf Rokney, Moti Baum, Sharon Amit, Ran Nir-Paz

**Affiliations:** 1 Department of Medicine, Hadassah Medical Organization and Faculty of Medicine, Hebrew University of Jerusalem, Jerusalem, Israel; 2 Government Central Laboratories, Ministry of Health, Jerusalem, Israel; 3 Department of Microbiology, Hadassah Medical Organization and Faculty of Medicine, Hebrew University of Jerusalem, Jerusalem, Israel; 4 Department of Medicine, Hadassah Medical Organization and Faculty of Medicine, Hebrew University of Jerusalem, Jerusalem, Israel

**Keywords:** *Staphylococcus aureus*, Polyclonal infection, Endocarditis

## Abstract

We present a case of bacterial endocarditis with both methicillin-sensitive and methicillin-resistant *Staphylococcus aureus*, which based on typing, originated from two distinct clones. Such a case may be misinterpreted by microbiology lab automation to be a monoclonal multi-drug resistant *Staphylococcus aureus*, while simple microbiology techniques will instantly reveal distinct clonality.

## Background

Bloodstream infection with *Staphylococcus aureus* (*S. aureus*) is a common occurrence in admitted patients. Such bacteremic events may represent one of many clinical scenarios from line infection, skin and soft tissue up to osteomyelitis or endocarditis, and may vary according to patients’ age [1, 2]. Several scoring systems were developed to identify patients with life-threatening infective endocarditis better [3]. Nevertheless, polymicrobial infections in the setting of *S. aureus* bacteremia are a less common occurrence, and infection with two subtypes/morphotypes of the same bacteria is considered an uncommon event [4]. Obviously, in the event of such multi-morphotype infections, a complicated and unanticipated clinical course could follow, resulting in a delayed antibiotic response. Thus, the importance of such an event is underlined by the prevalence of automated and molecular methods for the characterization of bacterial isolates, which may—in certain cases—fail to detect such occurrences.

## Case presentation

A 37-year-old homeless patient was admitted after being found lying in the street, weak but fully conscious. On arrival, the patient did not have any localizing symptoms but admitted to using intravenous (IV) heroin the same evening. One month prior to his current admission, he was observed for a small abscess in his right arm for which he refused percutaneous draining. The patient’s previous medical history includes untreated schizophrenia and glucose-6-phosphate dehydrogenase deficiency. During his current presentation, his vital signs were within normal limits and his physical examination was unremarkable except for a small abscess in his right arm. His blood tests were significant for a slightly elevated white blood cell count of 12.3 × 10^9^/L with a relative neutrophilia of 83.5%, hemoglobin 11.1 gr%, sodium 125 mmol/L and an elevated C-reactive protein (CRP) of 13.7 mg/dl. The rest of his blood tests, including ethanol, were normal. Urine toxicology was positive for cannabinoids and morphine. His chest X-ray was unremarkable, and electrocardiogram showed sinus tachycardia with no other abnormalities. On further exam, this finding on his right arm was determined to be an area of induration and cellulitis with no abscess. Early after his admission, he started suffering from a high fever, up to 40 °C. Blood cultures were obtained and antibiotic treatment with IV cefazolin 1000 mg three times daily (tid) was initiated. Concurrently, a new systolic murmur in the left sternal border was noted. His blood cultures were positive for methicillin-resistant *Staphylococcus aureus* (MRSA) with resistances profile that included clindamycin, erythromycin and methicillin. Treatment was changed accordingly to IV vancomycin 1000 mg twice daily (bid). The following day, another blood culture also came back positive for MRSA but with a difference in the resistances profile which now included chloramphenicol and methicillin. After 2 days, an additional blood culture came positive, but this time with methicillin-sensitive *Staphylococcus aureus* (MSSA), with resistances to clindamycin and erythromycin (Table 1).

**Table 1:** Antibiogram showing differences in blood cultures

Culture time	Organism	Chlora	Clinda	Tri-sul	Doxy	Eryth	Genta	Mino	Mupi	Oxa/methi	Rifa	Vanco
Day 1	*S. aureus*		R	S		R	S	S	S	R	S	S
Day 2	*S. aureus*	R	S	S		S	S	S	S	R	S	S
Day 4	*S. aureus*		R	S	S	R				S	S	

Breakpoints in lab are determined according to CLSI criteria

*Chlora* chloramphenicol; *Clinda* clindamycin; *Tri-sul* trimethoprim–sulfamethoxazole; *Doxy* doxycycline; *Eryth* erythromycin; *Genta* gentamycin; *Mino* minocycline; *Mupi* mupirocin; *Oxa/methi* oxacillin/methicillin; *Rifa* rifampicin; *Vanco* vancomycin

**Fig. 1:**
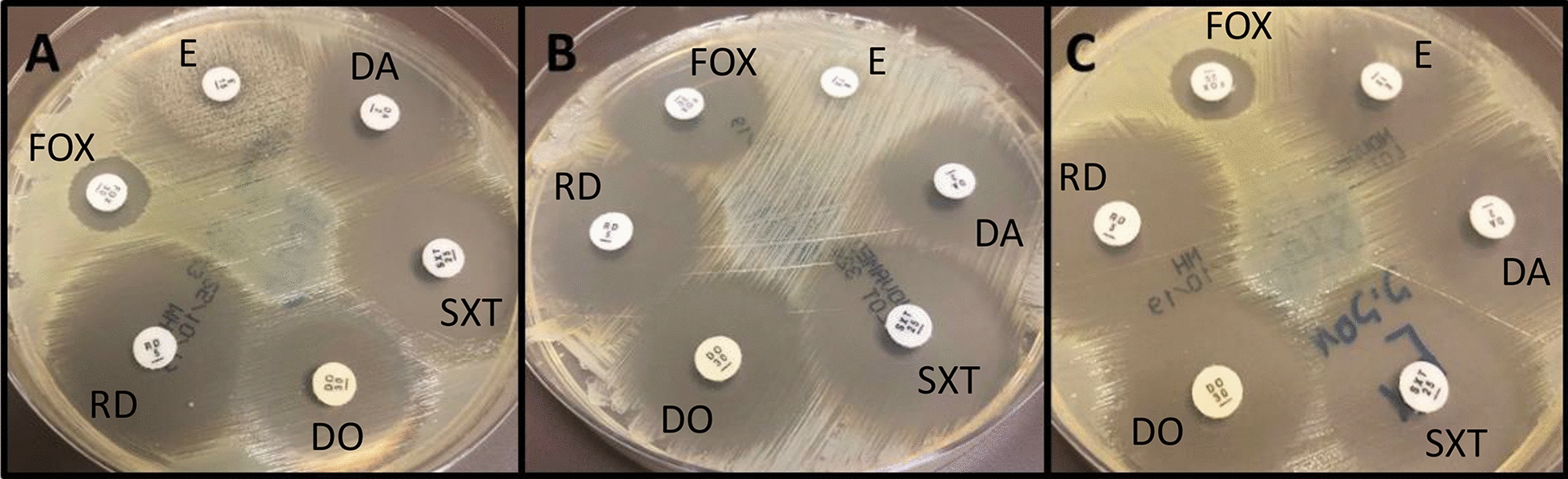
Two *Staphylococcus aureus* morphotypes isolated from a single blood culture. Muller–Hinton agar petri dishes with antimicrobial disks (E-erythromycin, DA–clindamycin, SXT–trimethoprim/sulfamethoxazole, DO–doxycycline, RD–rifampicin, FOX–cefoxitin) depicting the two morphotypes. **A** Mixed culture—two distinct zones are noted around the erythromycin disk. **B** Methicillin-sensitive *Staphylococcus aureus* (FOX sensitive) with inducible clindamycin resistance (positive “D-test”). **C** Methicillin-resistant (FOX resistant) *Staphylococcus aureus* sensitive to all other antimicrobials

This raised the question of a possible lab misinterpretation or a rare case of infection with two distinct *S. aureus* clones simultaneously. The lab was notified of the two possibilities and to our surprise, microbiological lab analysis revealed two distinct *S. aureus* isolates (Fig. 1). Further analysis at the *S. aureus* national reference center confirmed the presence of *mecA* in the MRSA isolate. In addition, the isolate was Panton–Valentine Leukocidin (*pvl)* positive, *spa* type t121 (repeat succession 11–19-21–17-34–24-34–22-25), and SCC*mec* type IV. The MSSA isolate was found to be *mecA* and *pvl* negative, and *spa* type t6605 (repeat succession 08–16-02–25-02–02-25–34-25).

Due to multiple positive blood cultures with both MSSA and MRSA, a new murmur, and a history of IV drug use, infective endocarditis was suspected. Rheumatoid factor and complement levels were within normal limits. Trans-thoracic echocardiography showed an echogenic mass of 10 × 7 mm on the tricuspid valve, attached to the septal leaflet, with at least moderate tricuspid regurgitation. Trans-esophageal echocardiography confirmed the previous findings and showed flail prolapse of the septal leaflet with a ruptured chordae tendineae and a severe, very eccentric jet of tricuspid regurgitation. According to the modified Duke criteria [5], a diagnosis of right-sided endocarditis was confirmed, with two major (persistent *S. aureus* bacteremia, vegetation) and two minor (IV drug use as predisposition, fever) criteria.

After the initiation of vancomycin antibiotic therapy, the MRSA stopped growing from the blood cultures within 2 days, while MSSA cultures were still positive for additional three more days. The subsequent blood cultures were negative. The patient completed 1 month of treatment with IV vancomycin. He continued treatment with trimethoprim–sulfamethoxazole 800/160 mg bid and ciprofloxacin 500 mg bid orally for another week and was discharged in general good health. The patient returned to the emergency department 6 and 7 months after discharge for various non-cardiac and non-infectious complaints. Three sets of blood cultures that were obtained on those presentations were sterile.

## Materials and methods

Multiplex real time polymerase chain reaction for the simultaneous detection of *mecA*, *mecC*, *pvl* and *nuc* gene, which serve as an internal amplification control, was performed as described previously [6, 7]. With the exception of *mecA* primers and probe sequences which used the CDC TaqMan probes for *mec*A [8]

Molecular typing of the isolate by *spa* typing and SCC*mec* typing was performed as described previously [9]. The *spa* typing analysis was performed using BioNumerics 7.6.3 software.

## Discussion

*Staphylococcus aureus* bacteremia was initially described over a century ago [10]. It is a lethal pathogen with high morbidity and mortality rates; 10–30% of patients diagnosed with *S. aureus* bacteremia will die [11]. The emergence of drug-resistant strains, such as MRSA, particularly community acquired-MRSA strains, exacerbate the phenomenon. In this case, our patient’s MRSA strain was of the t121 clone. This strain is more common in Africa, Asia and Europe, and accounts for 5% of MRSA isolates in these regions. Nevertheless, only 10% of t121 clones are methicillin resistant [12]. The patient’s MSSA strain was of *spa* type t6605, a subtype of ST398. ST398 was found to be both hospital and community acquired. It is presumed to be of livestock origin, and it shows almost universal erythromycin and clindamycin resistance [13], as in our case. While coagulase-negative staphylococci are known contaminants of blood cultures and polyclonal bacteremia was previously described [14, 15], analysis of cultures of *S. aureus* do not typically show polyclonal bacteremia. In one study, molecular analysis of the first blood cultures taken from 41 patients with *S. aureus* bacteremia and 21 bacteremia-associated catheter tip isolates revealed monoclonality in 100% of the cases. Nevertheless, rare cases of polyclonal *S. aureus* bacteremia were previously described. In an analysis of 122 MRSA isolates from a neonatal intensive care unit, two cases showed two genetically unrelated strains which were isolated from a single episode of MRSA infection [16]. Recently, a case of a 36-year-old woman with a history of IV drug abuse, suffering from endocarditis with isolation of two MSSA strains was described [4]. Similarly, our patient was an IV drug user and presented with a high fever. Considering these reports, it is not surprising that multiple strains of *S. aureus* were isolated in nasal carriers [17]. A mathematical model predicted that about 6.6% of *S. aureus* carriers host more than one strain [18].

To our knowledge, this is the first description of concurrent isolation of different molecular types of both MSSA and MRSA in a single episode of bacteremia. Although it was never described before, maybe it was just overlooked in most cases. It was previously argued that physicians have a cognitive bias regarding the diagnosis of infective endocarditis [19], and it may be one of the causes for the rarity of the diagnosis of polymicrobial endovascular infections. This case emphasizes that even though automated blood cultures in the microbiology lab have many advantages, in a case of an infection with different strains of the same species, there may be false negative results. The use of agar plates is still highly valuable in these cases. A high index of suspicion led to the diagnosis in this case, but there is a possibility that in some cases we miss the whole picture due to automation. High-risk populations such as IV drug users should always raise concern for polymicrobial infection, and clinicians must be alert.

## Data Availability

All data generated or analyzed during this study are included in this published article.

## Abbreviations

IV: Intravenous
MRSA: Methicillin-resistant *Staphylococcus aureus*
MSSA: Methicillin-sensitive *Staphylococcus aureus*
*pvl*: Panton–Valentine Leukocidin
*S. aureus*: *Staphylococcus aureus*
